# Community Health Worker Programs to Improve Healthcare Access and Equity: Are They Only Relevant to Low- and Middle-Income Countries?

**DOI:** 10.15171/ijhpm.2018.53

**Published:** 2018-07-01

**Authors:** Sara Javanparast, Alice Windle, Toby Freeman, Fran Baum

**Affiliations:** Southgate Institute for Health Society and Equity, Flinders University, Adelaide, SA, Australia.

**Keywords:** Community Health Workers, Primary Healthcare, Health Equity, Healthcare Access, High-Income Countries

## Abstract

**Background:** Community Health Workers (CHWs) are proven to be highly effective in low- and middle-income countries with many examples of successful large-scale programs. There is growing interest in deploying CHW programs in high-income countries to address inequity in healthcare access and outcomes amongst population groups facing disadvantage. This study is the first that examines the scope and potential value of CHW programs in Australia and the challenges involved in integrating CHWs into the health system. The potential for CHWs to improve health equity is explored.

**Methods:** Academic and grey literature was searched to examine existing CHW roles in the Australian primary healthcare system. Semi-structured telephone interviews were conducted with a purposive sample of 11 people including policymakers, program managers and practitioners, to develop an understanding of policy and practice.

**Results:** Literature on CHWs in Australia is sparse, yet combined with interview data indicates CHWs conduct a broad range of roles, including education, advocacy and basic clinical services, and work with a variety of communities experiencing disadvantage. Many, and to some extent inconsistent, terms are used for CHWs, reflecting the various strategies employed by CHWs, the characteristics of the communities they serve, and the health issues they address. The role of aboriginal health workers (AHWs) is comparatively well recognised, understood and documented in Australia with evidence on their contribution to overcoming cultural barriers and improving access to health services. Ethnic health workers assist with language barriers and increase the cultural appropriateness of services. CHWs are widely seen to be well accepted and valuable, facilitating access to health services as a trusted ‘bridge’ to communities. They work best where ‘health’ is conceived to include action on social determinants and service models are less hierarchical. Short term funding models and the lack of professional qualifications and recognition are challenges CHWs encounter.

**Conclusion:** CHWs serve a range of functions in various contexts in Australian primary healthcare (PHC) with a common, valued purpose of facilitating access to services and information for marginalised communities. CHWs offer a promising opportunity to enhance equity of access to PHC for communities facing disadvantage, especially in the face of rising chronic disease.

## Background

The World Health Organisation (WHO) defined community health workers (CHWs) as ‘members of communities where they work, selected by and respond to; are supported by the health system but not necessarily a part of its organisations; and have shorter training than professional workers.’^1^ The history of CHWs traces back to the 1970s and their introduction principally aimed to improve maternal and child health, and the management of common infectious diseases in settings with limited health workforce and low access to basic health services notably in low income countries.^2^ Many developing countries deployed CHW programs to tackle local health issues particularly in rural and remote areas. Examples of large-scale CHW programs are reported from countries such as Brazil, Iran, and Indonesia.^2-4^

In 1978, the WHO conference on primary healthcare (PHC) at Alma Ata explicitly cited CHWs as being one of the cornerstones of comprehensive PHC by providing basic health services, and contributing to achieving the key principles of community health and PHC: equity, community involvement, responding to local health needs, and inter-sectoral collaboration.^5^ The concept of a ‘task shifting approach’ (ie, shifting some healthcare tasks from medical and other practitioners to less specialised health workers), developed by WHO in 2008, reinforced the role of CHWs.^6^ Task shifting strategies were introduced to address the shortage of human resources for health, improve access, save costs, and meet local needs by expanding tasks undertaken by local and community health workforces.^6^ Task-shifting programs have, however, been criticised for being mainly focused on clinical tasks with insufficient recognition of the contributions CHWs can make to other aspects of comprehensive PHC such as health promotion and community development.^7^

More recently, the use of CHWs has attracted attention in some high-income countries where despite more developed health systems there are large inequities in healthcare access and outcomes amongst different population groups. Health inequity is defined as disparities between population groups that are avoidable, unfair, and unjust.^8^ Access to health services that are available, acceptable and affordable is a key, but not sufficient, component of health equity.^9^ Necochea et al consider CHWs as increasingly important in high-income countries alongside other health providers who deliver healthcare in homes and communities and facilitate access to PHC.^10^ The growing interest in CHWs in high-income countries is being driven by concerns about shortage in health workforce,^11^ and the escalating burden of chronic and complex diseases that is driving a significant increase in health services demand and costs in many developed countries.^12^ The American Public Health Association (APHA) has defined CHW as ‘a frontline public health worker who is a trusted member of and/or has a close understanding of the community served. This trusting relationship enables the CHW to serve as a link between health/social services and the community to facilitate access to services and improve the quality and cultural competence of service delivery.’^13^ The US Bureau of Labor has recognised CHW as an occupation^14^ and their potential to contribute to the health system has been highlighted in the Patient Protection and Affordable Care Act.^15^ According to the US Bureau of Statistics the employment of CHWs is projected to grow by 13% from 2014 to 2024, faster than the average for all other occupations in health system.^16^

Strong evidence exists on the contribution that CHWs have made to improving access to PHC, quality of care, and health outcomes in developing countries^2^ including reduced malnutrition rates,^17,18^ improved maternal and child health,^19^ prevention and management of HIV/AIDS,^20^ and management of infectious diseases.^21^ Furthermore, a recent study has shown CHW programs to be cost-effective in developing countries.^22^ A scoping review of contemporary literature on CHWs in the context of high-income countries has also suggested positive health outcomes in population groups experiencing disadvantage such as migrants, low socio-economic communities and Indigenous people.^23^ Most CHW literature in high-income countries comes from the United States and shows the significant role that CHWs play in engaging with patients and families and helping them to navigate the complex health and social systems.^2^ Literature suggests the effectiveness of CHWs in patients’ use of preventive services such as breast and cervical cancer screening among low-income and immigrant populations,^24,25^ the provision of culturally appropriate care, and health education and advocacy.^26^ CHWs have proven to have positive effects in chronic disease management including significant impacts on diabetes care, hypertension and cardio-vascular diseases^2^ and their clinical outcomes,^27^ increasing access and utilisation of PHC services, reducing hospital admissions and improving post-hospital care.^28^ A study assessing the impact of CHW outreach interventions on healthcare utilisation among African-American patients with diabetes showed a 40% decline in emergency room visits and 33% reduction in hospital admissions.^29^ In another study of primary care underuse among men in the United States, it was found that, because of a CHW intervention, care shifted from expensive inpatient care to less costly primary care services, with a return on investment of $2.28 per $1 spent on CHW intervention.^30^ Nevertheless, it is argued that assessing cost-effectiveness only in terms of health impact and return on investment fails to capture the total benefits of CHW programs in terms of their contribution to addressing community needs, and improved social inclusion and community empowerment.^31^ The role of CHWs in the provision of services beyond individual and clinical care has been reinforced through a study undertaken in Canada and suggests the significant role of CHWs in pre-natal health promotion outreach, community development and addressing social determinants of health among migrant and refugee groups.^32,33^ The success of CHW programs in other high-income countries provide evidence of their potential application in Australia.

Australia lags behind some other high-income countries in defining the scope of CHW interventions, and the utilization of CHWs to close the gap in healthcare access and outcomes among different population groups. There is an extensive body of literature on the wide gap in health status between aboriginal and non-aboriginal Australians.^34^ Critical to closing the gap in life expectancy (currently 11.5 and 9.7 years for males and females respectively)^35^ is improving aboriginal peoples’ access to culturally appropriate PHC^36^ and to address the ongoing effects of colonisation and racism in access to healthcare. There is also a large migrant and refugee population in Australia who lack English language skills, experience difficulties in navigating the health system, and suffer from discrimination, and racism.^37,38^

Despite international evidence on the effectiveness of CHWs, they have been largely overlooked by the Australian health system, including receiving very little research attention. The distinct but analogous aboriginal health worker (AHW) role is comparatively well recognised, understood and documented in Australia.^39^ A majority of AHWs hold certificates or diplomas, gained through registered training organisations.^40^ Some may have trained as an enrolled nurse^41^ and some pursue university training to enhance specific skills.^42^ A wide range of roles are identified for AHWs depending on the setting they work in and local needs. These include cultural brokerage roles^43^ (assisting aboriginal people and mainstream health services to interact more effectively by overcoming cultural barriers); clinical roles such as health checks and immunisation,^41^ and health education and health promotion including programs around child health, drug and alcohol issues, and healthy lifestyle programs.^44^ Literature points to the important role that AHWs play in improving cultural competency in healthcare settings^45^ and improved access to PHC.^46^

Despite the existing knowledge and experience on the role and contribution of AHWs to Australian PHC, there is a dearth of evidence on the scope of practice for the broader range of CHWs and the contributions they can make to improving health services and outcomes in the Australian context. Lessons from the AHW programs could inform and expand role for CHWs in Australia for other groups. Positioning CHWs as part of a coordinated workforce strategy offers an opportunity to enhance the performance and efficiency of the health system and improve population health equity and outcomes.^38^ The development of comprehensive health workforce policy and practice models to embed CHWs into the Australian PHC is impeded by a lack of evidence on existing CHW programs and their scope of practice. This study is the first that examines the scope and potential value of CHW programs in Australia and the challenges involved in integrating CHWs into the health system. It contributes to the knowledge of CHW programs in developed countries and potential for their integration in the formal health system as a strategy to reduce inequity in health access and outcomes.

## Methods

We employed two methods: (*a*) a review of academic and grey literature on CHW programs in Australia; (*b*) telephone interviews with a total of 11 stakeholders, including policy-makers, program managers and practitioners.

### Review of Academic and Grey Literature

A literature search of the following electronic databases was conducted: Scopus, Web of Science, ProQuest, and Informit. Since there is no definition of CHWs in Australia we used broader search terms including: community health worker/program/aide/role, aboriginal or indigenous health worker, lay health worker, peer educators, outreach worker, health advisor, ethnic health workers, primary health/primary healthcare.

More than 1000 results were returned, and these were filtered to remove duplicates, to adhere to the inclusion criteria and for relevance. The inclusion criteria were English language, published after 2005, and referring to a program/role in Australia. Documents identified as potentially relevant were retrieved in full text. Studies were included even if they were not written for the purpose of describing a program/role, but could provide relevant information from an observational or experimental study.

A further grey literature Google search was conducted using combinations of the following terms: community health worker, ethnic/multicultural health worker/program/ scheme, primary healthcare worker, women’s health worker, Medicare local, primary health network/PHN (current structure of PHC organisations in Australia). References cited in the included documents were examined, and links from relevant web pages were followed to identify further relevant material.

The final search result included published and unpublished reports and papers, journal articles, policy documents, position descriptions, and organisation and program websites. A total of 47 documents were found relevant to the study aims and were collated for review and analysis. Information was drawn from the literature to populate a table capturing aspects of the CHW role and organisational context. Table headings included program name and background information; location/region; community population they work with; recruitment and training; scope of practice; supervision; employment status; funding and sustainability and; evaluation/research findings. This review provided a picture of existing programs and their characteristics in Australia.

### Individual Interviews

A purposive sample of key informants was invited to participate in a semi-structured interview. Interview subjects were selected to gather the perspectives of a range of stakeholders from policy, management and practice, and relating to a range of community groups identified in the document review. We approached nineteen people who were involved in government structures relevant to health workforce issues and/or those who managed or practiced in specific CHWs programs identified during the document review. Participants were also identified through the researchers’ networks and knowledge of the Australian health system, and selected based on their experience or responsibility for CHW programs. The organisations initially contacted included Australian Federal and two State Departments of Health, Australian Medical Association, Nursing and Midwifery peak body, Rural Medical peak body, College of General Practitioners (GPs), PHC organisations and services, and migrant health services and councils. Eleven people agreed to be interviewed. Interviews covered: a definition of CHWs, perceptions about CHWs, benefits of CHWs in PHC, barriers and enablers to using CHWs, and the risks or disadvantages to having CHWs as part of the health workforce. Telephone interviews were conducted between June and September 2016. Interviews took 51 minutes on average, ranging from 28 to 67 minutes. Table provides details on study participants including positions, the nature of programs being managed by the program managers and the roles being played by the practitioners.

**Table:** Study Participants and the Programs in Which They Were Involved

**Participants (5 Males and 6 Females)**	**Number**	**Program Involved**
Policy-maker	2	- One from Federal Department of Health - One from State Department of Health
Program manager	6	- Chronic disease program - Multicultural child health promotion program - Aged care and dementia program - BCE program - Bi-cultural health program - CHW program for PWIDs
Practitioner	3	- Nurse (Australian Nursing and Midwifery Federation) - Medical practitioner (Rural Doctors Association of Australia) - Nurse/Coordinator (migrant health service)

Abbreviations: BCE, Bilingual Community Educator; CHW, community health worker; PWIDs, people who inject drugs.

An interview schedule was developed by the research team and included questions to draw out information on participants’ understanding, experiences and perceptions of CHWs in PHC, at both practice and policy levels. Interview schedules were modified slightly depending on whether the interviewee had direct experience of a CHW program/service, or whether they were speaking from a policy perspective. Eleven telephone interviews were conducted, audio recorded, and transcribed for analysis. NVivo 11 (QSR International) was used to develop a coding framework and analyse transcripts. One interview was double-coded by two members of the research team, indicating a high degree of agreement between the coders. Emerging themes and the coding framework from all interviews were discussed by the team in a workshop to ensure consensus in data interpretation.

## Results

In general, there was a paucity of literature describing CHW types and roles in Australian PHC system. Much of the literature was not written for the purpose of describing the workforce, and tended to be trials or evaluations of certain tasks within the role, or new programs utilising the role. The grey literature yielded additional information about various roles, which tended to come from organisational plans or annual reports, or from position descriptions. Very little evaluation of CHW programs in Australia was published in either the peer-reviewed or grey literature. Of 47 documents reviewed, 29 documents were on the role of AHWs, 13 targeted non-English speaking people, 2 targeted injecting drug users, 1 on people with dementia, and 2 not-specified. Figure shows a summary of CHW roles derived from the literature search.^41,47-86^

**Figure:**
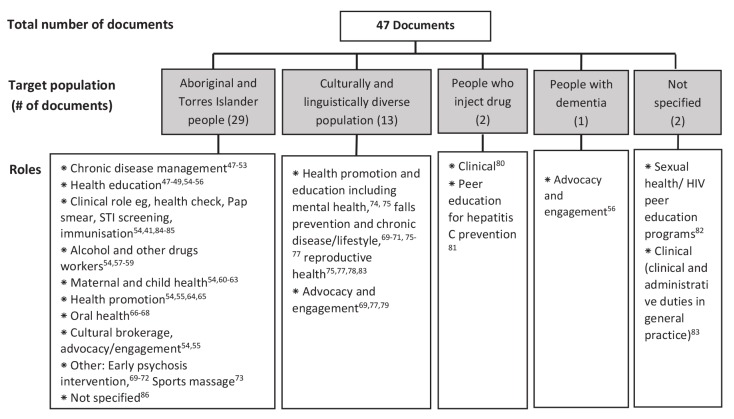
Summary of the CHWs Roles and Positions in Australian PHC. Abbreviations: CHW, community health worker; STI, sexually transmitted infection; PHC, primary healthcare. Note: Some papers may be counted more than once in a category, if the position performed a number of roles.

A broad range of roles for CHWs were identified reflecting the considerable variation in the strategies employed by CHWs, the communities they serve, and the health issues they address. The literature also revealed various terms used for CHWs. These include Indigenous Health Worker, Multicultural/Bicultural/Lay cultural health worker, Multicultural liaison officer, Bilingual/Peer/Community health educator, personal care worker, and community navigator.

The literature showed that the target population that CHWs work with are mainly aboriginal people, and culturally and linguistically diverse (CALD) people, including refugees. The most illustrative example is the AHW model which first emerged with aboriginal community controlled health services in the 1970s. In recent times there have been moves to recognise and support these important health workers through registration and the establishment of a professional body. The significant representation of AHW programs in this research is not unexpected, in that it probably represents their relatively high prevalence, the research and policy priority placed on closing the large gap between aboriginal and non-aboriginal Australians’ life expectancy, and their more formal recognition and organisation in the Australian PHC workforce (compared with other CHW programs). There are currently 606 ‘aboriginal health practitioners’ registered with the Australian Health Practitioner Regulation Agency (AHPRA).^41^ AHWs fit within the American Public Health Association’s broad definition of CHWs and they play a well-recognised role in the context of aboriginal community controlled health services, so their inclusion in literature review was important. However, a study undertaken by Health Workforce Australia suggests that the magnitude of the contribution of AHWs to improving access to PHC services for aboriginal people is not well understood and suggest that clear definition of their roles, structure of their training and recognition of AHWs as core components of the PHC workforce by other health professionals and health policy-makers are required.^86^

There is also some literature on ethnic health workers in Australia who address access and language barriers for new migrants and refugees.^79^ A bi-lingual CHW program in Queensland has been shown to improve access to quality healthcare for ethnic communities through raising awareness, building capacity, bridging communities to healthcare providers and addressing the social determinants of health.^79^ Several documents referred to programs for particular population groups on the basis of health status, for example people who inject drugs, older people with dementia or victims of genital mutilation. These communities are all linked by experiencing disadvantage and often marginalisation from mainstream society in Australia.

### Knowledge and Definition of Community Health Workers

Some interview participants had difficulty in defining the term CHW. There was confusion about whether CHWs were a particular role, or an umbrella term to describe health professionals who worked in a community setting (as opposed to hospitals). Some acknowledged that they could not provide a clear definition. There were varying interpretations of what constitutes a CHW, depending on the sector and the degree of experience with CHWs. Those interviewees with direct experience of working with CHWs had generally a clear understanding of CHWs which aligned with the American Public Health Association’s definition.^13^ Those who did not have direct experience of working with CHWs had difficulty in defining their roles, and tended to define them according to their qualification. This lack of clarity about CHW roles tied in with lack of appreciation of their value and niche. Such a perspective is shown from the interview with a policy-maker who commented:

*“My understanding is that community health worker [CHW] is a role that is used more in developing countries to try and get some basic health services where there is no other qualified health worker. I question what the value of it may or may not be in the Australian context, given we already have a plethora of different roles in Australia”* (Policy-maker).

A theme that was directly stated and also observed in the interview data was that the definition of CHWs can be influenced by the definition of ‘health.’ Those who defined health broadly with respect to social determinants, also defined CHWs relatively broadly, and saw greater value in CHWs. Whereas those with a medicalised, ‘health services’ perspective of health, struggled to define CHWs as they don’t tend to fit in the hierarchical view of ‘health’ as a service. A distinction was framed in the interview data as either services applying episodic, narrowly focussed services on the one hand, and those responding to systemic factors that influence health on the other:

*“I’ve been thinking a lot about social determinants of health and how maybe a lot of services don’t speak that language. So their own definition of health is limited and so they don’t see the broader aspects of life contributing to someone’s wellbeing. They’re not working more globally on contributing factors to peoples’ health, they’re really just doing band aid fixing and not doing anything systemic, not doing anything radical to improve peoples’ health.”* (Program manager).

### Community Health Worker Rles and Responsibilities

Data from the literature showed a wide range of roles and responsibilities for CHWs, across a range of contexts, as shown in Figure. AHWs performed a number of roles, including some quite specific clinical or education functions. This inclusion of clinical functions may have been possible because of the support and evolution of the AHW role over a number of years, and the sometimes remote contexts in which some AHWs are employed, where there is a dearth of other health practitioners.^48^

Those CHWs working with CALD communities tended to have less clinical roles, instead focusing on education and advocacy/engagement roles including:

- facilitating connection/relationship strengthening between community members and health service providers;
- supporting staff cultural competence and cultural appropriateness of services;
- building health literacy;
- facilitating access to community groups for research purposes; and
- seeking community input into development of programs/services.^69,83,87^

The degree of ‘contact’ and ‘personalisation’ of the engagement and advocacy differed across programs – some involved directly working with individuals to introduce them to a health service, others involved meeting with groups or representatives of community members. Community education as a function of CHWs was a prominent theme, with CHWs often specifically trained to deliver certain key messages. Examples include understanding the Australian healthcare system,^79^ or women’s reproductive health.^75,77^ Related to this, several interviewees mentioned the importance of outlining clear scope and boundaries, to ensure CHWs stay within what they are allowed to cover in an education session.

In some of the CHW roles discussed in interviews there were multiple tasks undertaken by CHWs from clinical support to more advocacy and engagement functions. Similarly, there was often overlap between providing education and a more personalised advocacy/support component. For example a multicultural health worker, who in response to an identified issue in a particular community, liaises with respective GPs about how to improve access to services, as well as coordinating and delivering community group information sessions. The value of individual CHWs spanning a number of roles, and performing a range of functions was identified by several interviewees. This flexibility was considered important in being able to meet and adapt to the needs of the community.

### Community Acceptability and Value of Community Health Workers

From our interview data we observed that the common characteristic of CHWs in the various activities and roles is being a person who is *trusted* by the community. This is beneficial both in terms of the relationship with the individual CHW, but also in the influence they can have in building the cultural safety of the service.

Trust was seen as a particularly important benefit of CHWs in marginalised communities such as CALD populations and people who inject drugs. The lack of trust between aboriginal and Torres Strait Islander people and government systems and services is well established,^63^ and in this respect the role of the AHW assists in bridging this trust gap between community and services.

While there was some discussion of building trust through credibility, via clear role definition and professional recognition, the prominent factor that enabled trust was *connection* with the community. A range of factors that contributed to connection between CHWs and the community were identified. For example familiarity, that often comes through being a member of the community, or having ‘*lived experience*’ and understanding the social and economic environment of the community. This was also framed in terms of being on the same ‘level’ as the community, in contrast to the social, educational and financial circumstances of particularly the medical profession, but also in terms of the ‘status’ of health professionals:

*“Well they understand the social and the economic environment within which the patients exist, and often the medical and nursing staff don’t actually comprehend the level of disadvantage and the cultural and social barriers with accessing care”* (Medical Practitioner).

Tied in with this connection through familiarity is the sense of the CHW being *non-judgemental*, and helping clients to feel comfortable and able to disclose information. This is particularly important for marginalised communities such as people who inject drugs. Several positive examples were provided in relation to this community, illustrating the improved healthcare access and outcomes a trusted CHW can enable, through earlier identification and intervention of health issues.

*“…if they didn’t feel comfortable opening up about that [inappropriate methadone use], then it would be really hard to give them the right treatment, so I think that couldn’t have happened unless you had community health workers [CHW]”* (Program Manager).

The CHW was perceived as being ‘*on the side’* of the community and able to advocate for them. This was particularly emphasised for remote communities, where nurses and other health professionals are often *‘agency’* or *‘fly in, fly out,’* and have little or no continuity with the community. Familiarity was also tied in with proximity – interviewees felt the CHW needed to be based in the community where the need is, rather than a ‘central’ location.

*Communication and language* were also identified as critical factors in building trust between CHWs and the community, both in terms of communicating in languages other than English, but also in using particular terminology or slang used by a particular community group, and at the appropriate literacy level. As one interviewee commented *“the vibe,*” or cultural appropriateness of communication is a related important factor. Listening was also identified as an important communication skill that helped to build understanding and connection: *“It’s a curiosity and it comes through sensitivity”* (Program Manager). As with any health service, confidentiality was also identified as important to building trust. It was emphasised that CHWs offer a level of engagement and relationship beyond that of language translation, and in some cases were employed alongside translators, with distinct roles.

Interviewees also indicated that in order to be accepted by the community, the CHW needed to have the ability to understand how people define their own health, rather than having pre-determined ideas about what people need to be healthy. This notion relates to the earlier discussion of CHWs’ role and purpose aligning with a social conception of health, rather than a ‘medicalised,’ service-oriented view of health.

It was acknowledged that recruiting appropriate people is important in achieving trust in CHWs. There can be diverse ethnic and religious backgrounds within a particular community group, which can make it hard to find a CHW who is widely acceptable. One interviewee indicated that in these situations, an ‘*outsider*’ may be more appropriate. Age and gender are also important considerations in terms of acceptability, for example there are restrictions in some aboriginal and Torres Strait Islander, and some CALD communities on discussing certain issues with members of the opposite sex or different generations.

*“Female health workers, which is the majority of them, often find the males won’t talk to them because it’s men’s business. Again, that’s a cultural issue that we need to understand when we’re employing”* (Medical Practitioner).

Because of their community connection, trust, and ability to understand both ‘sides’ of the health service delivery relationship, CHWs can serve as a ‘bridge’ between the community and the health system. This linking function was well articulated by one interviewee:

*“So one of the main things that they do is they act as a sort of two-way cultural broker. They help clients understand about the health system, and they help us to understand about the cultural issues that are impacting on our clients”* (Program manager).

The individual support obtained from CHWs was also highlighted:

*“…being the go-between or the facilitator that makes the whole health experience a whole lot smoother”* (Nurse Practitioner).

Providing this ‘bridge’ can help to facilitate access to information or services, contributing to positive health outcomes, for example improved knowledge about health behaviour, and a corresponding reduction in soft drink consumption as a result of community education by a CHW. Another example was increased utilisation of antenatal care by CALD women, facilitated by CHWs. There was also anecdotal evidence of lower emergency department presentations in an area where there is a primary health service with CHWs, for people who inject drugs. CHWs can also facilitate links between services/organisations to build cultural competency, foster collaboration and facilitate access across the health and social services system.

### Training and Qualifications

In many countries, the training for CHWs has been seen as a predictor of successful implementation of CHW programs and the quality of services they provide^49^ with on-the-job training as the most common type of training in high-income countries.^54,88^ In Australia, unlike most other health professions, CHWs are not required to have formal qualifications or registration. Historically, CHWs have not had formal training, but some employers prefer a Certificate IV in PHC, a vocational level of post-secondary education, below the level of a university degree. The interviewees frequently noted there is no national consistency or standardisation for CHW training. There were differing views in relation to the qualifications of CHWs. Even among interviewees with direct experience with CHWs, some felt that formal education/qualifications could increase the capacity of CHWs, whereas others felt they were not necessary to the function of CHWs. In contrast, interviewees acknowledged that there are designated qualifications and formal, nationally credentialed registration for AHWs, who as a workforce group are better understood and recognised. This is largely due to the historical and well-established, strong and multiple roles AHWs play within aboriginal community controlled health services.^89^

The lack of formal qualifications makes ‘professional recognition’ difficult. Health professionals’ scope of practice is often determined by their qualifications, the lack of which adds to the uncertainty about CHWs’ role. Interviewees acknowledged that formal qualifications are highly valued in society, especially in government departments, but this was felt to be less so for NGOs. Categories’ of health workforce are often aligned with medical and technical aspects of health, rather than social and cultural, and as such CHWs are more difficult to categorise the relative lack of status possibly also reflects the disparate, hidden nature of the front-line work they do largely in marginalised, disadvantaged communities.

While the National Aboriginal and Torres Strait Islander Health Worker Association encompasses AHWs, the absence of a broader CHW peer network or professional association, as exists for other health professionals, contributes to CHWs having significantly less power in the health system, compared with other workforce groups.

The challenge of striking the right balance between formality and informality of CHW workforce qualification and training pathways was well acknowledged from the interviews. While increasing the requirements on qualifications and registration of CHWs could improve their recognition and status, one interviewee felt that the requirement for formal training risks excluding people from communities experiencing disadvantage who have strong connection with their community, from becoming a CHW. If training is formalised, it must be readily accessible to such people, and the increasing accessibility of technical and vocational education courses for CALD people was acknowledged as aiding their recruitment.

*“…to have this role really formally recognised, we need to be qualified and accredited, but can that then exclude really good people?”* (Program Manager).

Interview data showed what could be described as an interconnected vicious cycle: the lack of awareness of CHWs contributes to a lack of demand for the role/workforce, and poor employment prospects. This contributes to a lack of training options/places where ‘vocational’ training delivery is increasingly delivered by private providers.

### Funding and Sustainability

Funding constraints were seen to be a key limitation. Funding for CHWs comes from a range of sources, including Federal, State and local government, regional PHC organisations and non-government organisations. In some cases CHW roles are voluntary but most discussed in this study were paid positions. CHW services/programs are relatively insecurely funded, with contracts of one to three years, with on-going funding rare. The fact that CHWs usually work with communities facing disadvantage means there is unlikely to be a ‘market’ for these services to be provided on a private fee-for-service basis, and the role needs to be funded by public health services.

The insecure nature of funding was felt to impact on the sustainability of CHWs’ role in that it had implications for retention of staff, and also contributed to a lack of trust in the health system due to the short term, ‘project-ism’ approach:

*“Well, our system itself doesn’t allow us to really embed a program and get that sustainability, so therefore there’s a lack of enthusiasm with anything new because they think, well, how long’s this going to last. And you do get that - we’ve had this reform, we’ve changed this structure so many times, we start a project and then that finishes and then that service isn’t available anymore”* (Program manager).

Sustainability is also greatly influenced by factors other than funding. Whether the role is understood and valued is critical, and related to this is the relative lack of ‘status’ of CHWs that comes with their lack of formal qualification.

*“I mean, there are some challenges in relation to the valuing of the role within [the government department of] health, and the lack of a clear classification/qualification and role within the team, because they’re very easy to pick off”* (Program manager).

One interviewee talked about the benefits of clearly articulating the CHW role and its value as this helped to maintain the role over a long period of time. From the perspective of another interviewee from government, without direct experience of working with CHWs, this lack of recognition and status was seen as a benefit, in that CHWs have relatively little power for lobbying for wages, advocacy and expansion of role scope, and competing with other health professions:

“*the minute you sort of give them a title, call them a particular thing, they start advocating for all sorts of things and the states and territories then get terribly, terribly nervous and then go, ‘We can’t afford to pay them that much money” (Policy-maker).*

Consistent with this, the importance of having high level advocacy and being valued by decision makers were also seen as key determinants in the sustainability of CHW roles, and the vulnerability to the shifting priorities and foci of governments was acknowledged. It was felt that financial constraints limited the capacity for ‘additional’ aspects such as evaluation, as service delivery was a higher priority. This potentially leads to a vicious cycle, in that if a program is not well evaluated and its benefits clearly demonstrated, it is difficult to source ongoing funding for a group lacking power within the system. Interviewees noted that better evaluation of CHW programs would help to enhance their sustainability. In contrast to other examples of CHWs, AHWs seemed to have more recognition, funding allocation and program sustainability. This was largely due to the critical role that AHWs play in aboriginal health services.^89^

### Integration of Community Health Workers in the Health System

The integration of CHW programs into the nationwide PHC system through definition and recognition in healthcare planning was seen as crucial for improved effectiveness and sustainability.^90^ Recognition of the role of AHWs has been a key factor in their acceptance and integration within PHC multidisciplinary teams.^89^ Interviewees widely acknowledged that acceptance of and respect for CHWs by other health service providers were key factors in ensuring their successful integration in the health system. As discussed previously, appreciating and valuing the CHW role by other health providers and stakeholders is closely tied in with factors such as professional recognition and credibility, as well as organisational culture supporting a broader social definition of health. Concerns were raised about the issues of competition, ‘*territory’* and perceived threat from CHWs by other providers, in particular nurses. This is because nurses’ roles are potentially more at risk of being usurped by the less qualified, lower paid CHW. It may also be an issue of professional culture misalignment, with doctors and nurses generally having a background in a medical construct of health, whereas CHWs flourish best where health is understood in the context of its wider social determinants.

Again*, clarity of role scope* emerges as a key issue, in terms of being seen as a mitigating factor against the risk of competition and ‘turf’ issues between providers. If the CHW role scope is clearly defined and articulated, this can minimise concerns from other providers about encroaching into their role scope, both within an organisation and between organisations in a region.

*“I think as long as you make it really clear the Multicultural Health Worker is not doing the doctor’s role, is not doing the dietician’s role, is not doing a physio’s role - so we normally are quite cautious. So if we interact with any health services the first thing we do is that orientation to make sure that we are all clear who is doing what and how we can work together”* (Program Manager).

The importance of CHWs adhering to their scope in an education-focussed role, and not providing advice in the domain of another health professional was raised in relation to their acceptance by other health providers, both as a risk management consideration, and in terms of competition.

Some concerns were raised in the context of clinical aspects of CHWs’ work, around the risks associated with ‘delegating’ tasks to an unqualified worker. There were some views that the Australian health system is risk-averse, and that some might see CHWs as too risky. However, these concerns are based on an approach to CHWs as lower qualified workers to whom tasks can be shifted, rather than recognising their wider value in connecting with communities.

A number of other factors were identified as being important enablers of CHWs in PHC, that are common to most jobs, including good communication and systems, good teamwork and management, and the CHW being empowered with the appropriate autonomy to perform their role to its full scope.

## Discussion

This scoping study of CHWs in Australia is significant in being the first to provide an overall picture of existing CHWs in Australia, and to explore the main challenges that exist in the utilisation of CHWs as an effective health workforce strategy. This study indicates that the advances in the legislative, educational and research efforts that have been undertaken in the United States^2,91^ and the positive results in the role that CHWs can play to address health needs of vulnerable population groups could also be relevant to Australia. The findings of this Australian CHW study, although limited in scope, provide some promising results about their potential contribution to improving healthcare access and equity for some population groups including Indigenous communities, people from CALD populations, and people suffering from complex health conditions such as drug addiction. This is also consistent with findings from other studies in high-income countries that suggested the need for ‘better integration of CHW programs within the broader health system to enable their full potential to be realised.’^23^

The findings from this study shows that some key elements of CHW programs are highly relevant for disadvantaged groups of population in high-income countries. Trust, community connection, advocacy, and bridging communities to formal health systems are core to the value of CHWs and are critical in improving healthcare access and equity.^82^ Despite the variety of terms used in Australia, CHW programs discussed in this study have had a common, valued purpose of facilitating access to health services and information for communities which are vulnerable and marginalised, by providing a trusted link that can help to bridge cultures and disadvantage. Examples of CHW programs in this study have revealed the potential contribution that CHWs can play in health education and social determinants of health which is partly due to their good understanding of the social conditions within which patients live.

Our findings align with the literature that suggests a CHW can perform a broad range of health-related functions and link communities to the health system.^54^ A review of organisational interventions to improve access to PHC for vulnerable population groups in high-income countries has identified CHW programs as a key strategy in facilitating integration of health and social services which is relevant to the Australian context.^92^ Another study reviewing international innovations for improving access to PHC for vulnerable populations found CHW interventions were an important strategy to address some dimensions of healthcare access including acceptability and appropriateness.^93^ The findings from our study and also broader literature on CHWs in high-income countries especially the USA reveal that CHWs have the potential to play a critical role in closing access gaps in PHC.

There are, however, many challenges that high-income countries such as Australia face in the implementation of CHW interventions and their integration into the formal health system. There is no single definition for CHWs in Australia with no clarity on their role and scope of practice. The registration and formal training of AHWs in Australia is a good example of formalising a CHW program that is proven to be effective in improving aboriginal health outcomes. Improved career paths for AHWs and the organisational support they receive from the Community Controlled PHC service model could be a model for all CHWs in Australia.

Consistent with the literature from many high-income countries, studies that demonstrate the effectiveness of CHW programs are limited in Australia. While there were examples of favourable experiences from the perspective of those with first-hand knowledge of CHWs, low knowledge and awareness of CHWs, as well as lack of supporting evidence base in the literature hinder their wider adoption and integration. Robust evaluation of the outcomes and economic value of CHW models is required at the community, practice and policy level to underpin initiatives to support greater appreciation, utilisation and sustainability of CHWs in the Australian PHC workforce. As noted by Torres et al an ‘empowerment model’ is required to make CHWs more visible within the health system workforce.^94^ Policies with a focus on equity, and that recognise CHWs as vital partners in the health and human resources workforce will help to maximise their role in addressing health needs.^94^ Research and evaluations of CHWs need to also consider social benefits, improved community capacity, and equity gains as a result of CHW interventions.^23^ Our findings support the conclusions by Balcazar et al that ‘given the range of roles played by CHWs and the broad range of settings and health issues in which they work, a single-minded focus on something like the cost-effectiveness of a narrowly defined CHW intervention will not provide usable and sufficient evidence to help shape policy and program planning.’^91^

The lack of evidence on effectiveness of CHWs creates a vicious cycle of poor knowledge on program effectiveness and health impacts, thus leading to the poor policy and financial priority given to CHWs in health policy, further leading to poor program implementation, research and evaluation. Without breaking this cycle, CHW programs will likely remain a low priority.

Our study had some limitations. While the study gives an indication of the types of roles CHWs perform, it should be interpreted with caution, and not considered a proportionally representative picture of the CHW landscape in Australia, given the paucity of literature. There may also be a publication bias, in terms of ‘innovative’ and experimental initiatives being over-represented in the literature, as opposed to the ‘everyday’ practice of these types of roles being less likely to be documented, particularly in the peer-reviewed literature. Furthermore, the scope of the study did not allow for community/clients perspectives on CHWs, which is crucial in understanding their contribution to community health.

## Conclusion

With a stronger evidence base, and considered policy development, CHWs offer a promising opportunity to enhance equity of access to PHC for Australian communities who experience disadvantage and marginalisation, improve education and community development, and help to meet the challenges of workforce shortages, increasing healthcare demand, and burden of chronic and complex health conditions.

## Acknowledgmens

The Australian scoping study was funded by the Flinders University Seeding Grant. We acknowledge the participation of those who accepted to be interviewed and gave their time and shared their experiences and views with us.

## Ethical issues

Ethics approval was grant by the Flinders University Social and Behavioural Research Ethics Committee (SBREC).

## Competing interests

Authors declare that they have no competing interests.

## Authors’ contributions

SJ, FB, and TF designed the study. AW collected and coded the data. All authors contributed to data interpretation and analysis. SJ and AW co-led the writing of the paper. All authors commented on drafts of the paper and approved the final draft for submission.

## Key messages

**Implications for policy makers**

- Community health workers (CHWs) offer a promising opportunity to enhance equity of access to primary healthcare (PHC) and improved health equity in Australia. There are many lessons from the success of CHW programs in low- and middle-income countries that can be learned in the context of high-income countries
- Evidence on CHWs’ effectiveness in high-income countries is sparse and thus greater research support will assist to produce evidence to inform policies related to the integration of CHWs in health system.
- Better understanding of the role of CHWs by practitioners and program managers would improve their acceptance and their inclusion in multidisciplinary PHC teams.
- A close collaboration between researchers, health policy-makers and CHWs is essential to close the gaps in identifying research priorities, implement action research with CHWs, and translate research findings into policy and practice.

**Implications for the public**

Community health workers (CHWs) are proven to be very effective in improving access to healthcare and health equity particularly for population groups most in need. Although there is strong evidence on the role and contribution of CHWs to health outcomes and their integration into health systems in low- and middle-income countries, such evidence is sparse in the context of high-income countries. This study examined the range and role of CHWs in Australia and the policy and research gaps that need to be addressed to enhance their contribution to health access and equity. This is particularly crucial in the face of increasing prevalence of chronic disease and subsequent health system demand and cost.
